# Shedding Light into the Connection between Chemical Components and Biological Effects of Extracts from *Epilobium hirsutum*: Is It a Potent Source of Bioactive Agents from Natural Treasure?

**DOI:** 10.3390/antiox10091389

**Published:** 2021-08-30

**Authors:** Gunes Ak, Gokhan Zengin, Mohamad Fawzi Mahomoodally, Eulogio Llorent-Martínez, Giustino Orlando, Annalisa Chiavaroli, Luigi Brunetti, Lucia Recinella, Sheila Leone, Simonetta Cristina Di Simone, Luigi Menghini, Claudio Ferrante

**Affiliations:** 1 Physiology and Biochemistry Research Laboratory, Department of Biology, Science Faculty, Selcuk University, 42250 Konya, Turkey; akguneselcuk@gmail.com; 2 Department of Health Sciences, Faculty of Medicine and Health Sciences, University of Mauritius, Réduit 80837, Mauritius; f.mahomoodally@uom.ac.mu; 3 Department of Physical and Analytical Chemistry, Campus Las Lagunillas S/N, University of Jaén, E-23071 Jaén, Spain; ellorent@ujaen.es; 4 Department of Pharmacy, Botanic Garden “Giardino dei Semplici”, Università degli Studi “Gabriele d’Annunzio”, via dei Vestini 31, 66100 Chieti, Italy; giustino.orlando@unich.it (G.O.); annalisa.chiavaroli@unich.it (A.C.); luigi.brunetti@unich.it (L.B.); lucia.recinella@unich.it (L.R.); sheila.leone@unich.it (S.L.); disimonesimonetta@gmail.com (S.C.D.S.); claudio.ferrante@unich.it (C.F.)

**Keywords:** *Epilobium hirsutum*, oenothein B, myricetin, antioxidants, antiproliferative effects, gene expression, bioinformatics

## Abstract

*Epilobium hirsutum* is extensively used as a traditional remedy in folk medicine, especially against prostate inflammation. Therefore, we evaluated the chemical profiles and biopharmaceutical potentials of different extracts of *E. hirsutum* aerial parts and roots. Metabolomic, antioxidant, and enzyme inhibitory profiles were investigated. Human prostate cancer PC3 cells were exposed to the extracts to evaluate antiproliferative effects. Gene expression and bioinformatics analyses were performed to investigate anti-inflammatory mechanisms. Oenothein B and myricetin were prominent compounds in the extracts. In scavenging/reducing assays, the methanol, infusion, and methanol/water extracts exhibited similar activities. We also observed the reduction of PC3 viability occurring following exposure to methanol and methanol/water extracts. According to bioinformatics analysis, myricetin was predicted to interact with COX-2 and TNFα. The interaction between TNFα and oxo-dihydroxy-octadecenoic acid was predicted as well. Intriguingly, the gene expression of COX-2 and TNFα was reduced in PC3 cells after exposure to methanol and methanol/water extracts. These effects were paralleled by the decreased gene expression of IL-8 and NFkB and the inhibition of PGE2 release. Therefore, the present findings suggest the potential use of *E. hirsutum* for the management of the burden of inflammation and oxidative stress occurring in lower urinary tract diseases, including prostatitis.

## 1. Introduction

In the past decade, people have been looking for new health-promoting compounds from natural sources. Among the natural sources, plants and plant products are the most important [1]. For example, the determination of antioxidant properties of plant extracts is an index of the efficacy against oxidative stress, which is associated with chronic inflammatory and degenerative diseases [2]. In addition to their antioxidant properties, the enzyme inhibitory, toxic, and anticancer properties of plant extracts have been attracting interest in the scientific community [3,4,5]. With this in mind, scientists are increasingly relying on plants as inspiration for new active ingredients that could be developed in the context of the pharmaceutical industry. Overall, this phenomenon is referred to as the green revolution, and new studies, especially on wild plants that have not yet been investigated, have made a significant contribution to this. However, the possible toxic properties of plants and their synergetic and antagonistic interactions should not be forgotten.

The genus *Epilobium* belongs to the Onagraceae family and comprises more than 200 species worldwide [6]. The members of the genus are known by different local names such as fireweed, wickopy, and niviaqsiaq [7]. *Epilobium* species have been widely used in traditional medicine. For example, *E. angustifolium* and *E. hirsutum* are known as yaki otu and are used to treat prostate diseases in Turkey [8,9]. Moreover, *E. hirsutum* is used to stop bleeding [10] in the treatment of gastrointestinal problems, menstrual disorders, and sleeping problems [11,12,13,14]. In addition, the young leaves of the *Epilobium* species are often used as food and feed [11,15]. To confirm the ethnobotanical uses of the species, several pharmacological and biochemical studies on the members of the genus have been performed [13,16,17,18,19,20,21,22]. In addition to these pharmacological studies, the presence of biologically active compounds including tannins (especially ellagitannins), myricetin, quercetin, and several phenolic acids have been reported in phytochemical studies [18,23,24,25,26,27].

In light of the above information, in the present work, we aimed to unravel the relationship between the chemical components and biological properties of various extracts (ethyl acetate, methanol, methanol/water and infusion) from two parts (aerial parts and roots) of *E. hirsutum*. The chemical characterization of the tested extracts was assessed using the HPLC-ES-MS^n^ technique. The intrinsic biological properties of the extracts were evaluated in terms of antioxidant and enzyme inhibitory effects, whereas the *Artemia salina* (brine shrimp) and human prostate cancer PC3 cell line were exposed to the extracts to define the biocompatibility limits and the antiproliferative effects, respectively. Finally, the anti-inflammatory properties of the extracts were studied through bioinformatics analysis and the evaluation of the gene expression of different pro-inflammatory biomarkers, namely TNFα, COX-2, VEGFA, IL-6, IL-8, NFkB, and TIMP1. The obtained results will shed light on the rational phytotherapy use of *E. hirsutum,* especially against inflammatory diseases in the prostate.

## 2. Materials and Methods

### 2.1. Plant Collection and Extract Preparation

*Epilobium hirsutum* L. samples were collected in the city of Erzurum (Karabıyık village, Turkey; GPS coordinate: 40°08′22″ N, 41°56′16″ E; altitude: 2220 m), in August (collection date: 20 August 2020). The plants were authenticated by a plant taxonomist (Dr. Evren Yıldıztugay) in the Science Faculty, Selcuk University, Konya, Turkey, and voucher specimens (Voucher number: GA-20-002) were kept at the herbarium of the above-mentioned faculty. The plant materials (about thirty samples) were collected in the same population.

The plant materials were packed in plastic bags. Thereafter, the aerial parts and roots were separated, and they were immediately dried in the shade for ten days (25 ± 2 °C). During the drying process, the temperature, humidity, and air flow were controlled to avoid the effects of the phytochemicals in the plants. After the drying process, the dried plant materials were ground using the laboratory mill in the same week. The powdered plant materials were stored in a well-ventilated place in the dark (20 °C). The plant materials were extracted immediately to avoid changing the chemical profile in the plants. After the powder process, the extraction of the plant materials was completed in one week. 

Regarding the extraction procedure, the samples were extracted with different solvents, namely ethyl acetate, methanol, methanol/water (70%, *v*/*v*), and water. To obtain the ethyl acetate, methanol, and methanol/water extracts, the plant samples (10 g) were macerated with 200 mL of methanol for 24 h at room temperature. Then, the extracts were filtered and evaporated. As for the water extracts, the fruit materials (10 g) were kept in 200 mL of water (boiled) for 15 min. The obtained water extracts were filtered, and a lyophilization process was conducted to remove the water. The obtained extracts were stored at 4 °C until experimentation.

### 2.2. Total Phenolic and Flavonoid Content

The Folin–Ciocalteu and AlCl_3_ methods were performed to evaluate the total amount of phenolic and flavonoid content as per our previous publications. Standard compounds (gallic acid equivalent: GAE for phenolic and rutin equivalent: RE for flavonoid) were also tested to explain the contents in the tested extracts [28,29].

### 2.3. Instrumentation

Chromatographic analyses were performed with an Agilent Series 1100 HPLC system with a G1315B diode array detector (Agilent Technologies) and an ion trap mass spectrometer (Esquire 6000, Bruker Daltonics) with an electrospray interface. Separation was performed in a Luna Omega Polar C_18_ analytical column (150 × 3.0 mm; 5 µm particle size) with a Polar C_18_ Security Guard cartridge (4 × 3.0 mm), which were both purchased from Phenomenex. Detailed chromatographic conditions are available in Fernández-Poyatos et al. [30].

### 2.4. Determination of Antioxidant and Enzyme Inhibitory Effects

The methodology used to determine the antioxidant capacity was performed by using different test systems including phosphomolybdenum, metal chelation, reducing power (FRAP and CUPRAC), and free radical scavenging (DPPH and ABTS). The results of the assays were explained as standard equivalents, namely Trolox (TE) and EDTAE (EDTA). As for the enzyme inhibition abilities, several enzymes, including cholinesterases, α-amylase, α-glucosidase, and tyrosinase, were selected. For each assay, standard compounds (galatamine, acarbose, and kojic acid) were used to explain the results. All of the details of the antioxidant and enzyme inhibition assays were reported in our previous paper [31]. The entire analysis was performed in triplicate, and the results are presented as mean ± SD. In order to identify the differences between the extracts, one-way analysis of variance (ANOVA) followed by Tukey’s test was performed. The level of significance was set at *p* < 0.05. The statistical analysis was conducted with XLSTAT 2016 software.

### 2.5. Artemia salina Lethality Test

The cytotoxicity limit of the extracts in the range 0.1–20 mg/mL were evaluated through the brine shrimp *Artemia salina* lethality bioassay, as previously reported [32]. The experiments were conducted in triplicate.

### 2.6. Cell Culture and Viability Test

The effects of the extracts (100–300 µg/mL) on human prostate cancer PC3 cell viability were determined through the 3-(4,5-dimethylthiazol-2-yl)-2,5-diphenyltetrazolium bromide (MTT) test. In the same concentration range, cells were treated with the extracts for evaluating prostaglandin E2 (PGE2) level (pg/mL) in cell supernatants via radioimmunoassay [33]. In parallel, the gene expression of pro-inflammatory biomarkers was evaluated as well. The experimental conditions of the cell culture were fully described in our previous paper [34].

### 2.7. Gene Expression Analysis

Gene expression of TNFα, COX-2, VEGFA, IL-6, IL-8, NFkB, and TIMP1 was conducted as previously reported [35]. Briefly, after extraction through the TRI Reagent, the total RNA was reverse transcribed using a High-Capacity cDNA Reverse Transcription Kit (ThermoFischer Scientific, Waltman, MA, USA). Gene expression was determined by quantitative real-time PCR using TaqMan probes obtained from ThermoFischer Scientific (Waltman, MA, USA). β-actin was used as the housekeeping gene. The data analysis was conducted with the Sequence Detection System (SDS) software version 2.3 (ThermoFischer Scientific, Waltman, MA, USA). A detailed description of the experimental protocol is reported in a previous paper of ours [35].

### 2.8. Statistical Analysis

The experimental data related to in vitro studies were analyzed through ANOVA followed by the Newman–Keuls post hoc test. The GraphPad Prism software was employed for the statistical analysis. *p* < 0.05 was considered statistically significant.

## 3. Results and Discussion

### 3.1. Total Phenolic and Flavonoid Content

In the current research, the total contents of phenolic and flavonoid in the tested extracts were evaluated through colorimetric assays. As it can be seen from Table 1, in the extracts from the aerial parts, the highest level of the total phenolics was observed in the methanol extracts, with a value of 254.55 mg GAE/g, followed by infusion (211.37 mg GAE/g), methanol/water (202.97 mg GAE/g), and ethyl acetate (43.52 mg GAE/g). In the tested roots, the extracts can be ranked as methanol/water (156.27 mg GAE/g) > infusion (152.64 mg GAE/g) > methanol (109.16 mg GAE/g) > ethyl acetate (32.52 mg GAE/g). Regarding the total flavonoid content, the extracts from the aerial parts contained a higher content of total flavonoids than the root extracts. The methanol extract from the aerial parts was noted as being the richest in total flavonoid content, with a value of 87.66 mg RE/g. The lowest level of total flavonoids was found in the methanol/water (2.41 mg RE/g) and methanol (2.54 mg RE/g) extracts of the roots (*p* > 0.05). These findings are in agreement with the literature data. For example, it was reported by Ege and colleagues [12] that the total phenol content in the macerated water extracts from the aerial parts of *E. hirsutum* was 225 mg GAE/g. Similarly, this value was reported by Dzhafar and colleagues [7] as 206.3 mg GAE/g in the ethanol-based lyophilized extract. In another study by Pourmorad et al. [36], however, the total phenolic content in the methanol extract of *E. hirsutum* was 92.12 mg GAE/g, therefore lower than that showed in our study. In addition, the total phenolic content for the members of the genus *Epilobium* was reported by several authors [11,16,21]. The observed differences between these studies could be explained by geographical (altitude, soil characteristic, etc.) and climatic conditions (rainfall, dryness, etc). [37,38,39]. In recent reports, the Folin–Ciocalteu test does not reflect the true content of phenols [40]; therefore, the results of this test should be confirmed by other analytical techniques such as HPLC-MS or NMR to allow a real comparison. For these reasons, the tested extracts were characterized using the HPLC-ESI-MS^n^ system.

### 3.2. HPLC-ES-MS^n^ Analysis

The characterization of the phytochemicals was conducted with HPLC-ESI-MS^n^ using the negative ion mode. Identification was performed using analytical standards—citric acid, coumaric acid, ferulic acid, gallic acid, kaempferol, and quercetin—as well as bibliographic information. As an example, the base peak chromatogram of the methanolic extract of aerial parts is shown in Figure S1 (Supplementary Material). The characterization of the compounds in all of the extracts is shown in Table 2. Compounds were numbered according to their elution order, with the same numbering being maintained in all extracts.

Compound **1** was tentatively characterized as a disaccharide (HCl adduct) due to the neutral loss of 162 Da (341→179) and the characteristic fragments of hexoside moieties (*m/z* 179, 161, 119, 113 and 101) [41]. The most plausible structure is a diglucoside.

Compound **2**, with deprotonated molecular ion at *m/z* 195, exhibited the base peak at *m/z* 129 and other fragment ions at *m/z* 177 and 159. This fragmentation has been previously reported for gluconic acid in *E. angustifolium* L. extracts [16].

Compounds **3** and **5** exhibited the same fragmentation pattern, corresponding to (iso)citric acid. The distinction between both isomers was performed by analyzing the analytical standard of citric acid.

Compounds **4** and **11** presented the same molecular ion and similar fragmentation patterns. This fragmentation pattern is consistent with oenothein B, a dimeric ellagitannin that has been reported in *E. angustifolium* [23].

Compound **7** was identified as gallic acid through comparison with the analytical standard. In addition, several derivatives were also characterized. Compounds **6** and **8** were characterized as digallylglucose isomers, which have been previously reported in *E. augustifolium* [25]. With additional 152 Da (gallic acid), compounds **15** and **16** were characterized as hexahydroxydiphenoyl-digalloyl-glucose and trigalloylglucose, respectively, as reported in *Epilobium* species [42].

Compound **10**, with [M-H]^−^ at *m/z* 311, showed tartaric and caffeic acids at *m/z* 149 and 179, respectively. It was identified as caftaric acid [43]. To our knowledge, this compound has not been previously reported in *Epilobium* species.

Compound **13** suffered a neutral loss of 132 Da (pentoside) to yield ferulic acid at *m/z* 193, so it was identified as a ferulic acid pentoside.

Compound **14** was tentatively characterized as roseoside (vomifoliolglucoside or drovomifoliol-*O*-*β*-*D*-glucopyranoside) based on bibliographic information [44]. This compound has not been previously reported in *Epilobium* species.

Compound **26** was identified as myricetin due to the [M-H]^−^ at *m/z* 317 and fragment ions at *m/z* 179 and 151. Three myricetin glycosides (compounds **17**, **18**, and **19**) were characterized based on the neutral losses of 132 Da (pentoside), 146 Da (deoxyhexoside), and 162 Da (hexoside). Similarly, compounds **20**, **22**, **23,** and **24** were characterized as quercetin glycosides (quercetin aglycone at *m/z* 301), and compounds **21**, **25,** and **27** were characterized as kaempferol glycosides (kaempferol aglycone at *m/z* 285). The presence of myricetin, kaempferol, and quercetin glycosides has been previously reported in *Epilobium* species [24].

Compounds **28** and **29** were tentatively characterized as saponins due to the similar fragmentation pattern reported for these compounds in other plant species [45].

Compounds **31** and **33** were characterized as the oxylipins oxo-dihydroxy-octadecenoic acid and trihydroxy-octadecenoic acid based on bibliographic information [46].

### 3.3. Quantification of Phytochemicals

We calculated the relative contribution of all of the compounds using the area normalization method (Table 3 and Table 4). Peak areas of each compound were obtained using the precursor ion [M-H]^−^ (Extracted Ion Chromatograms). Then, the relative contribution (in percentage) of each compound was calculated, and the heat map (the darker the color, the higher the abundance) was constructed. There were some differences between the extracts of the aerial parts and roots. The main compounds found in the extracts of the aerial parts were compound **11** (Oenothein B)—except in the EA extract—and compounds **17**, **18,** and **19** (myricetin glycosides). On the other hand, oenothein B was prominent in all of the root extracts, except in the EA extract, where oxylipins (compounds **31** and **33**) were the main contributors to the extract composition.

In the aerial parts, flavonoids accounted for 60–70% of all compounds (only 40% in the infusion extract); most of them were myricetin and its glycosides. However, in the roots, the contribution of flavonoids was very scarce, and oenothein B represented between 43% and 80% of all compounds, except in the EA, where oxylipins accounted for more than 50% of the detected compounds.

The production of phytochemicals in the plant metabolism is a dynamic process [47,48] and depends on several factors, including the vegetative period, collection year and time, and geographic locations [49,50,51,52]. Several studies have shown that both total and individual phenolic compounds have even changed in the same species due to geographical factors [37,39,53]. In addition to geographical factors, the amount of rainfall or drought between years can significantly influence the phytochemical composition of plant extracts [54]. For example, several researchers have reported that the production of secondary metabolites (especially the amount of phenolics) has increased against drought stress [55,56]. The increased content of phenolics can help to inhibit the oxidation of cells under stress conditions [57]. In this sense, the differences in *E. hirsutum* observed in previous studies could be explained by the factors mentioned above.

### 3.4. Antioxidant Properties

The evaluation of the antioxidant abilities of plant extracts could be an important signpost on the way from nature to the pharmacy shelf. In this sense, we investigated the antioxidant properties of the *E. hirsutum* extracts using different chemical approaches, including the quenching of free radicals, reducing power, and the chelation of transition metals. The results are presented in Table 5. DPPH and ABTS radicals are widely used to assess the quenching of radicals by plant extracts. In the tested aerial part extracts, the greatest radical scavenging ability was observed in the methanol extract (798.89 mg TE/g for DPPH and 1382.08 mg TE/g for ABTS). However, in the root extracts, the most active extract was methanol/water (317.72 mg TE/g for DPPH and 552.33 mg TE/g for ABTS). In both the aerial parts and the roots, the ethyl acetate extracts showed the weakest abilities on these radicals. Reduction power assays examine the transformation of Cu^2+^ to Cu^+^ and Fe^3+^ to Fe^2+^ in CUPRAC and FRAP assays, respectively. In these assays, the infusions obtained from both the aerial parts (1819.57 mg TE/g for CUPRAC and 756.63 mg TE/g for FRAP) and the roots (1065.96 mg TE/g for CUPRAC and 424.87 mg TE/g for FRAP) demonstrated the best abilities. The lowest abilities in both parts were also recorded in the ethyl acetate extracts. The phosphomolybdenum reaction involves the conversion of Mo (VI) to Mo (V) by the antioxidant compounds in the acidic pH. The assay has gained interest in recent studies because it is simple and does not need specific equipment. In the extracts from the aerial parts, the superior ability was demonstrated by methanol extract, with a value of 6.80 mmol TE/g followed by the infusion (4.72 mmol TE/g), methanol/water (4.25 mmol TE/g), and ethyl acetate (3.11 mmol TE/g) extracts. As for the root extracts, the methanol, infusion, and methanol/water extracts had similar abilities (*p* > 0.05). The chelation of transition metals is considered to be one of the most important antioxidant mechanisms that is able to stop the production of hydroxyl radicals in the Fenton reaction. In the present study, among the tested extracts, the methanol extract of the aerial parts had the strongest chelating ability, with a value of 63.31 mg EDTAE/g followed by infusion of the aerial parts (58.19 mg EDTAE) and the roots (45.22 mg EDTAE/g). When all of the antioxidant results were combined, the results were correlated with the chemical components of the tested extracts. For example, the methanol, methanol/water, and infusion extracts contained higher amounts of phenols and were also most active in the antioxidant assays. As a further attraction, these extracts were rich in oenothein B and myricetin, which are known to be effective antioxidants [58,59]. For example, oenothein B is a unique class of ellagitannis, and its conformational structure with multiple OH- groups has exhibited remarkable biological activities including antioxidant, anticancer, and immunomodulatory effects [60]. Taken together, the observed antioxidant abilities of the tested extracts can be attributed to the presence of these components. In addition, several authors have examined the antioxidant capacities of the members of the genus *Epilobium,* including *E. hirsutum* [11,13,21,24,58], and in accordance with our presented study, significant antioxidant effects were observed with higher concentration of phenolics, particularly ellagic acid, oenothein B and its derivatives, and myricetin.

### 3.5. Enzyme Inhibitory Properties

In recent studies, the prevalence of non-infectious global diseases is increasing day by day, and urgent precautions are needed to control the prevalence of these diseases. In this context, researchers are seeking to find alternative and effective treatment strategies for managing these diseases. With the growing human population, synthetic compounds are still key players in these strategies [61]. However, since most of them have toxic properties, alternative compounds, especially those from natural sources, are gaining a lot of interest in place of these synthetic ones. Among them, natural enzyme inhibitors play an important role in the treatment of the above-mentioned diseases. These natural compounds could inhibit the actions of enzymes associated with different chronic inflammatory and degenerative diseases [62,63,64,65,66]. Therefore, natural extracts and compounds are often tested against enzymes such as cholinesterases, α-amylase, α-glucosidase, lipase, and tyrosinase as pharmaceutical targets.

In this regard, *E. hirsutum* extracts were tested against cholinesterases, amylase, glucosidase, and tyrosinase, which are deeply involved in Alzheimer’s disease, type-2 disabetes, and hyperpigmentation, respectively. The results are tabulated in Table 6. In AChE inhibition, the methanol extract of the aerial parts had the best inhibitory ability with a value of 4.48 mg GALAE/g followed by methanol/water (2.76 mg GALAE/g) and ethyl acetate (2.69 mg GALAE/g). The infusion of the aerial parts was not active on AChE. In the root extracts, the ability was in the following order: ethyl acetate > methanol > methanol/water > infusion. Taking into account the BChE inhibition, the strongest abilities were shown by the ethyl acetate extracts (4.72 mg GALAE/g for the aerial parts and 5.18 mg GALAE/g for the roots) in both parts, whereas the methanol extracts exhibited the best inhibitory actions against tyrosinase, with the values of 106.68 mg KAE/g (in aerial parts) and 98.31 mg KAE/g (in roots). However, the methanol extract of the aerial parts statistically had a similar efficacy compared to the methanol/water extract (*p* > 0.05). As for the α-amylase inhibitory effect, the methanol extracts from both parts displayed superior inhibitory potential, while the lowest enzyme inhibitory effects were obtained from the infusions. Interestingly, different results were observed in the α-glucosidase inhibitory assay. The most potent ability was detected in the infusion of the roots, with a value of 1.96 mmol ACAE/g. However, the methanol/water and infusion from aerial parts were not active on α-glucosidase. Overall, the methanol extracts from the tested parts were more active than other extracts. The observed findings could be explained by the presence of chemical components including oenothein B and myricetin. Our observations are consistent with previous studies that reported the significant inhibitory effects of these compounds [67,68,69,70,71]. In addition, remarkable enzyme inhibitory effects have been demonstrated for other plants belonging to the genus [11,21,58]. However, a comparison with our results is difficult because of different methods of expression, including the IC_50_ values or the percentage of inhibition.

### 3.6. Toxicological and Pharmacological Evaluation

**Figure 1:**
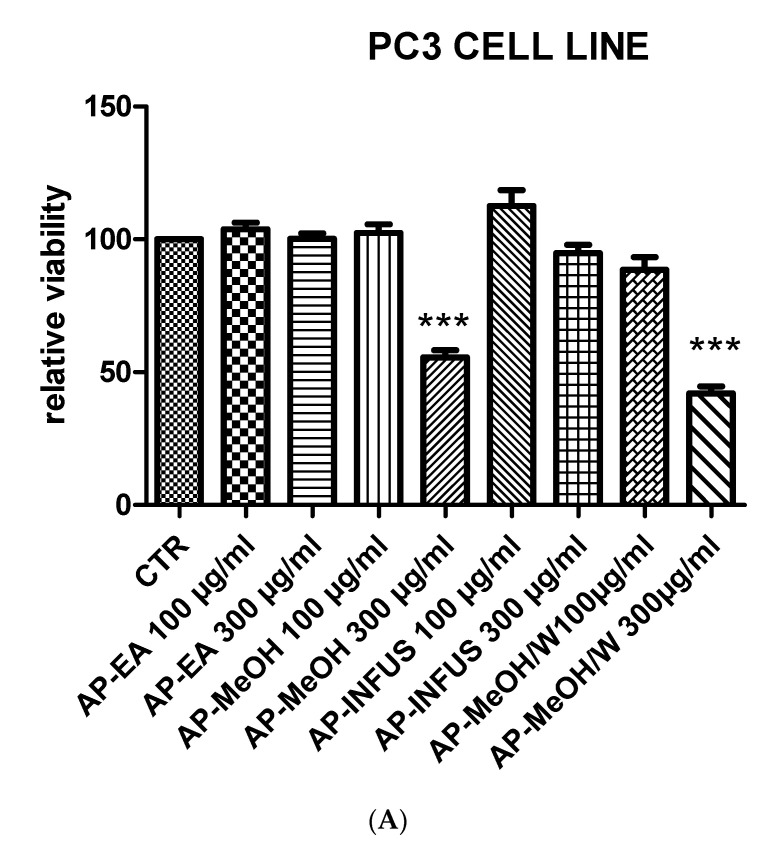

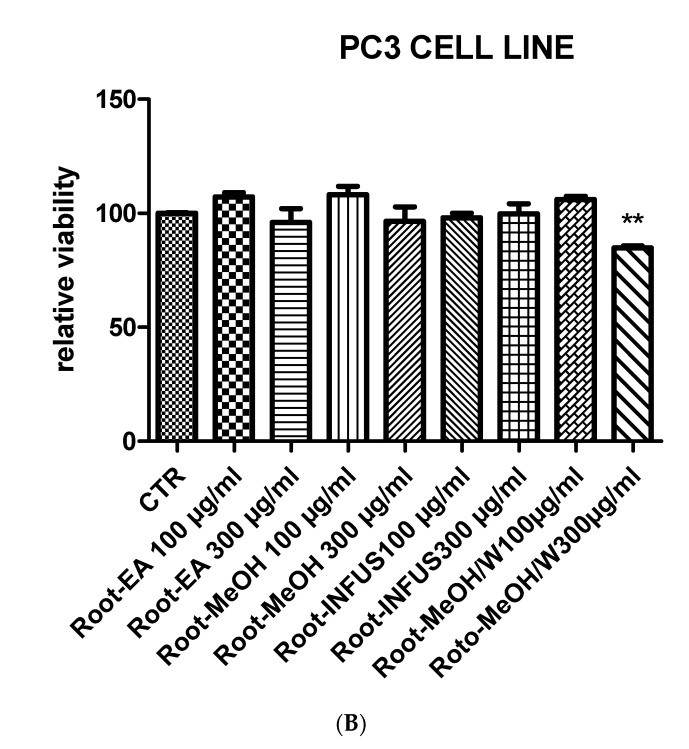
Antiproliferative effects induced by the methanol (MeOH), ethyl acetate (EA), water (infusion), methanol/water (MeOH/W) extracts of the aerial parts (AP) (**A**) and roots (**B**) from *Epilobium hirsutum* (100–300 µg/mL). ANOVA, *p* < 0.0001, ** *p* < 0.01, *** *p* < 0.001 vs. CTR (Control) group.

**Figure 2:**
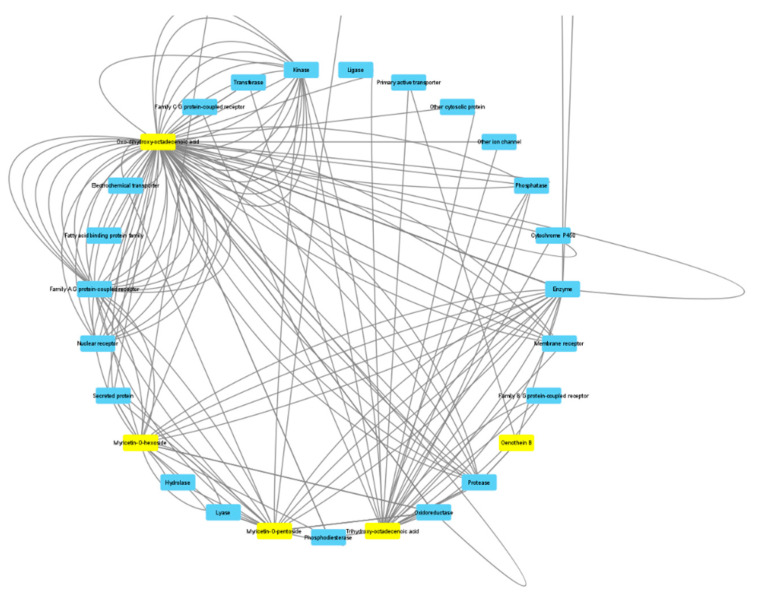
Target analysis of components underlying the putative interactions of oenothein B, myricetins, oxo-dihydroxy-octadecenoic acid, and trihydroxy-octadecenoic acid with a wide number of proteins, among which are oxidoreductases and secreted proteins, which are also involved in oxidative stress and inflammation. Proteins targeted by extracts were predicted using the bioinformatics platform SwissTargetPrediction, and the network pharmacology analysis was conducted through the software Cytoscape (Version 3.8).

**Figure 3:**
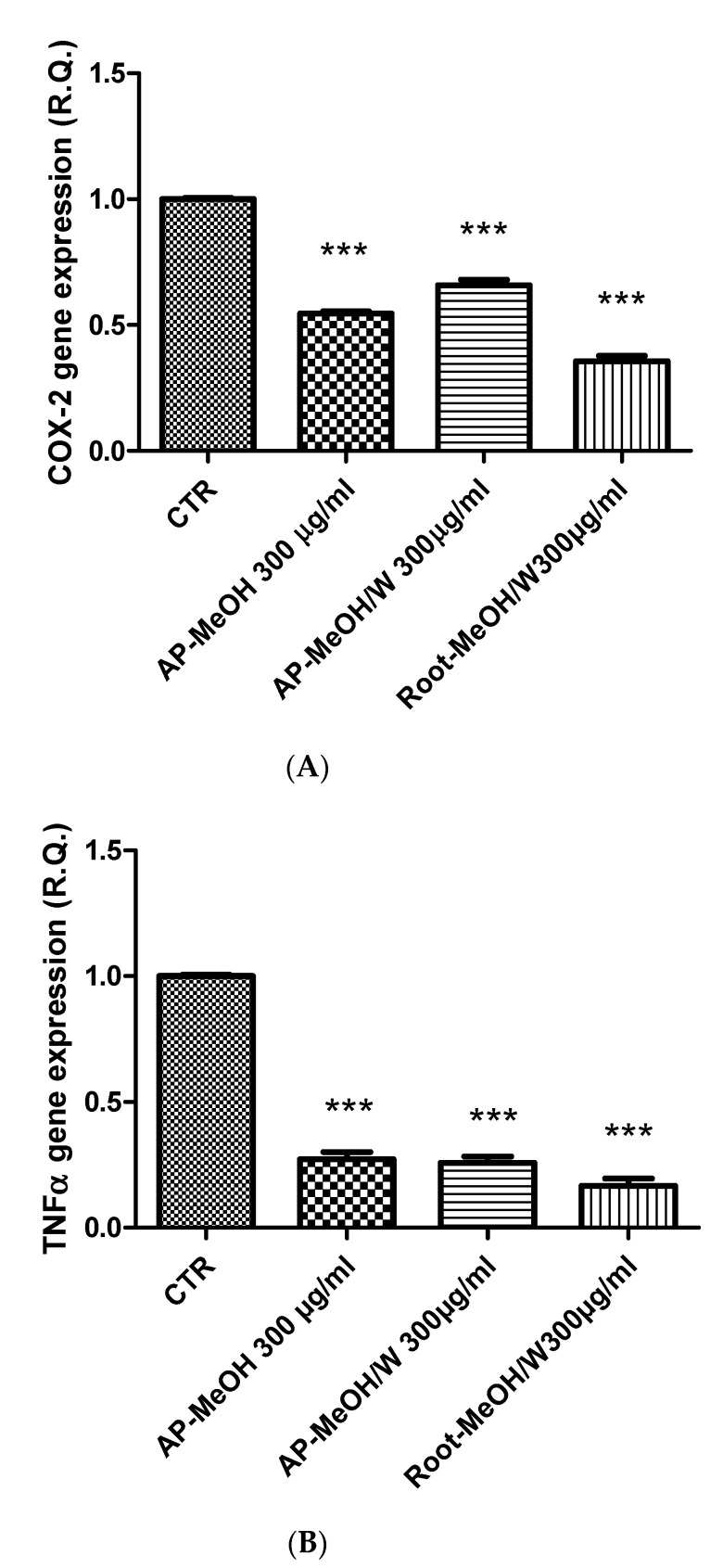
Inhibitory effects of COX-2 (**A**) and TNFα (**B**) gene expression induced by the methanol (MeOH) and methanol/water (MeOH/W) extracts of aerial parts (AP) and roots from *Epilobium hirsutum* (100–300 µg/mL). ANOVA, *p* < 0.0001, *** *p* < 0.001 vs. CTR (Control) group.

**Figure 4:**
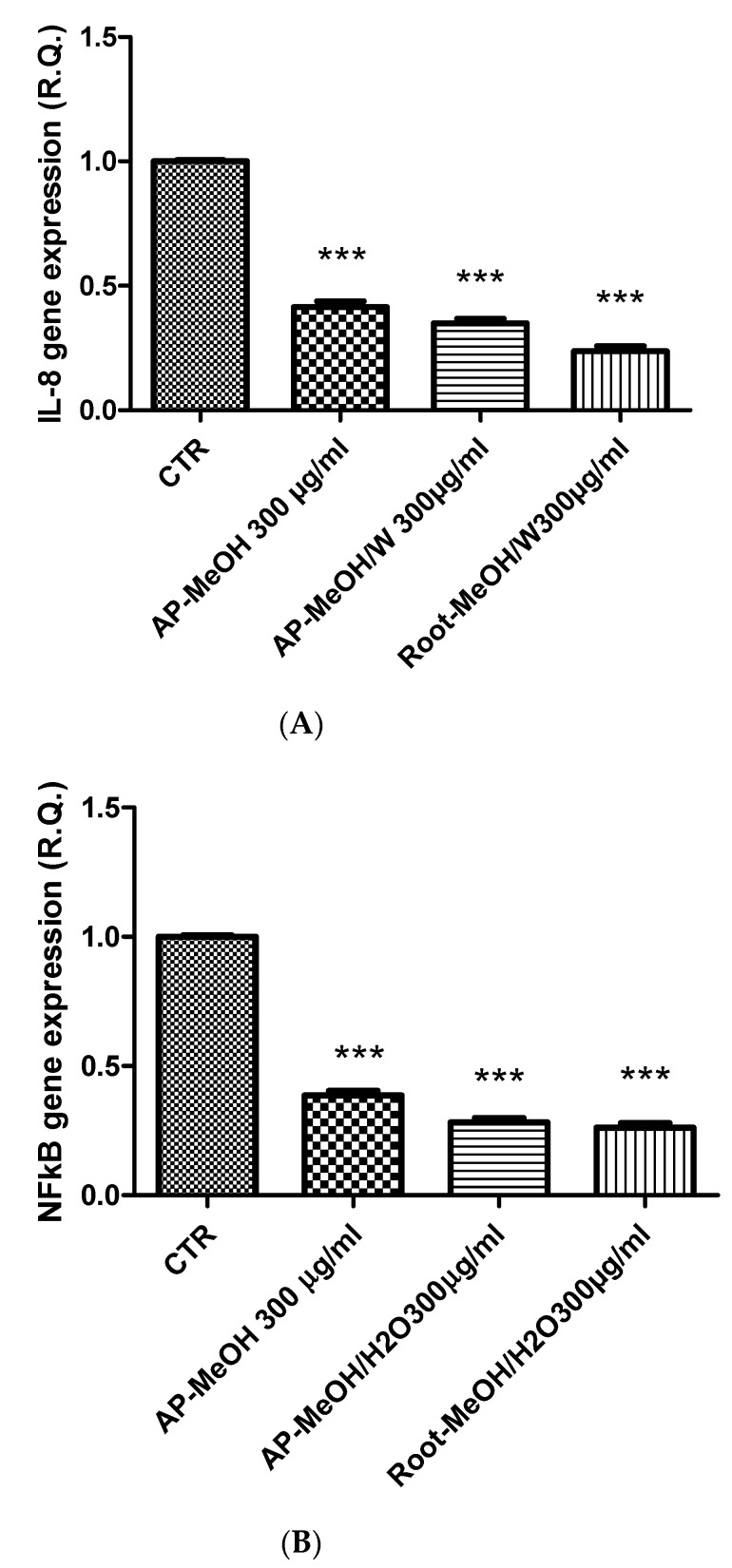
Inhibitory effects of IL-8 (**A**) and NFkB (**B**) gene expression induced by the methanol (MeOH) and methanol/water (MeOH/W) extracts of aerial parts (AP) and roots from *Epilobium hirsutum* (100–300 µg/mL). ANOVA, *p* < 0.0001, *** *p* < 0.001 vs. CTR (Control) group.

**Figure 5:**
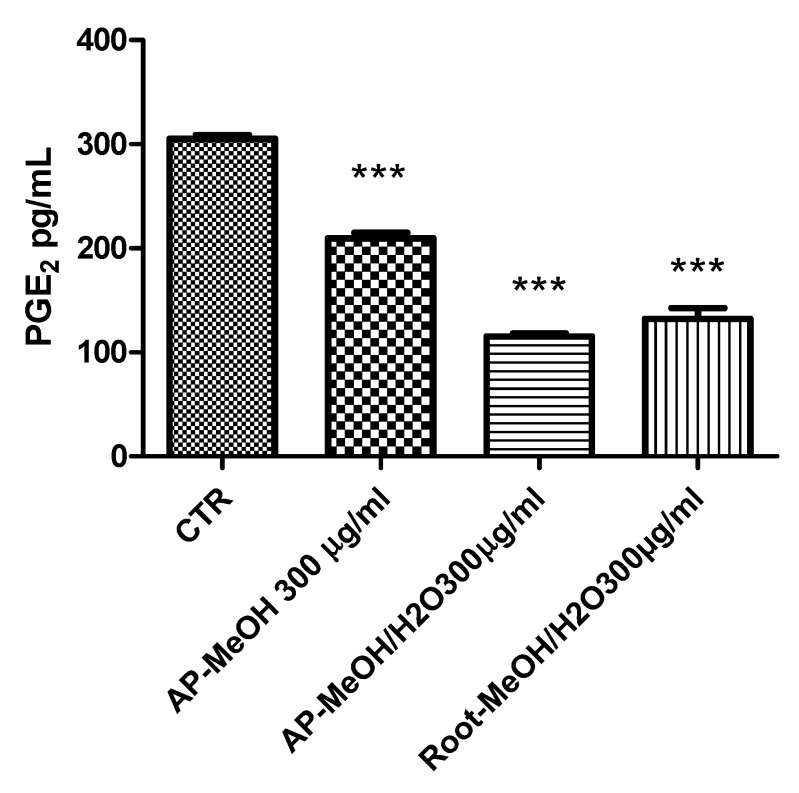
Inhibitory effects of PGE2 release induced by the methanol (MeOH) and methanol/water (MeOH/W) extracts of aerial parts (AP) and roots from *Epilobium hirsutum* (100–300 µg/mL). ANOVA, *p* < 0.0001, *** *p* < 0.001 vs. CTR (Control) group.

**Figure 6:**
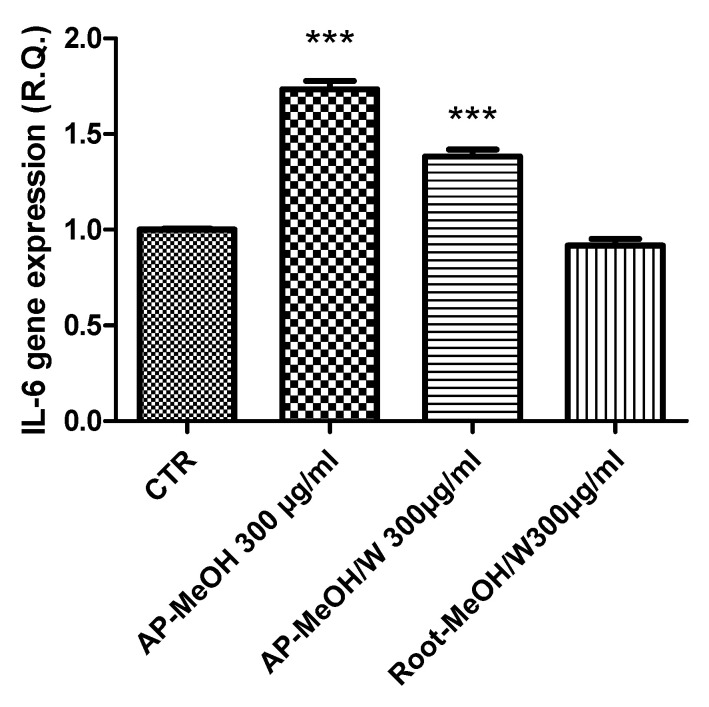
Stimulatory effects of IL-6 gene expression induced by the methanol (MeOH) and methanol/water (MeOH/W) extracts of aerial parts (AP) and roots from *Epilobium hirsutum* (100–300 µg/mL). ANOVA, *p* < 0.0001, *** *p* < 0.001 vs. CTR (Control) group.

**Figure 7:**
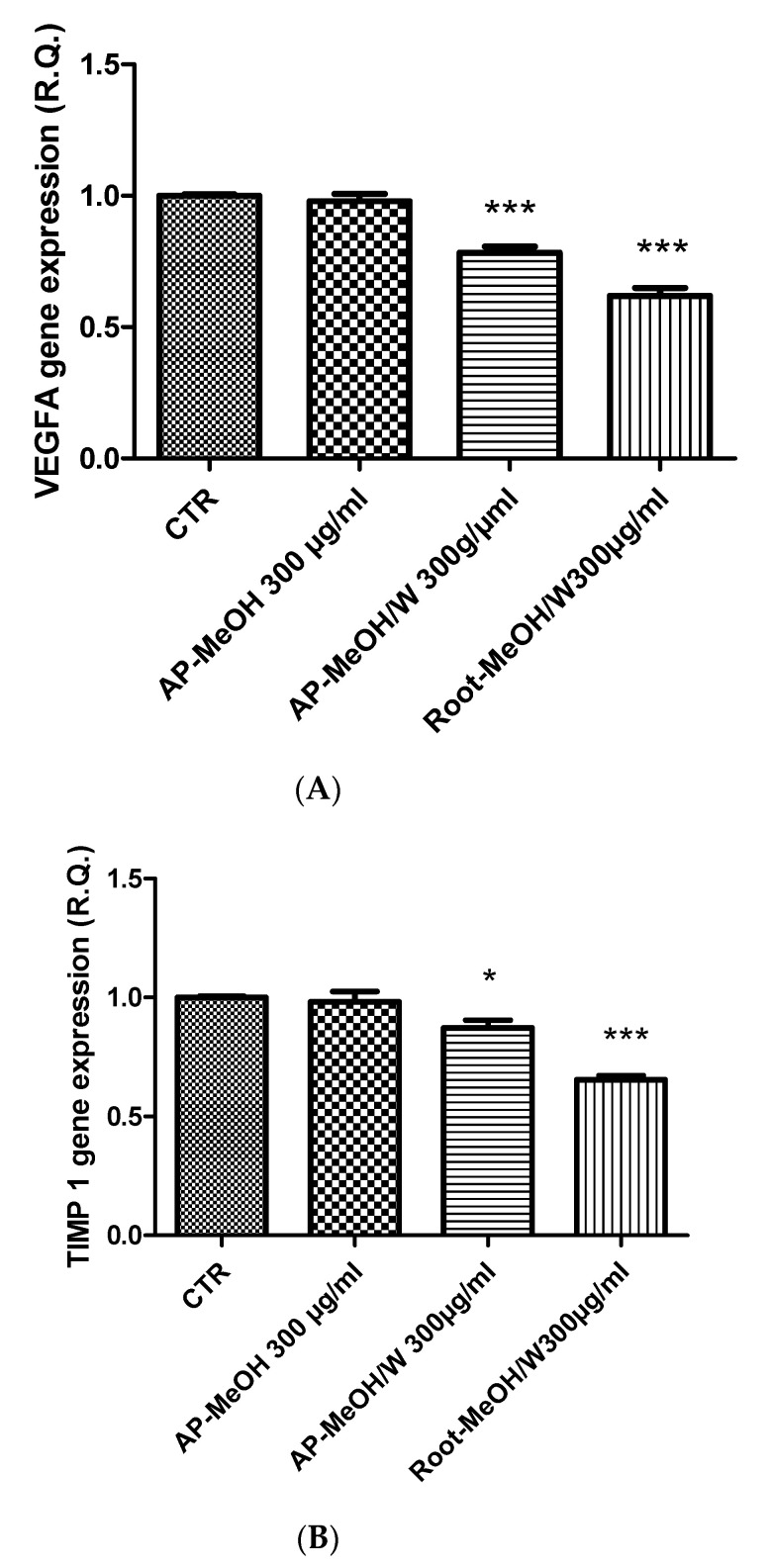
Inhibitory effects of VEGF (**A**) and TIMP1 (**B**) gene expression induced by the methanol (MeOH) and methanol/water (MeOH/W) extracts of aerial parts (AP) and roots from *Epilobium hirsutum* (100–300 µg/mL). ANOVA, *p* < 0.0001, *** *p* < 0.001, * *p* < 0.05 vs. CTR (Control) group.

**Figure 8:**
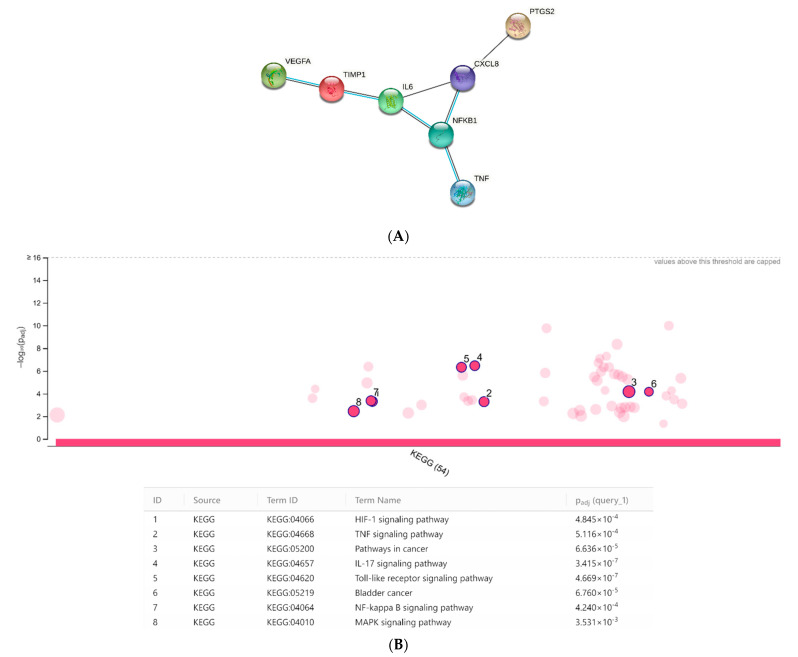
Protein–protein interactions (**A**) and KEGG analysis (**B**) conducted through the bioinformatics platforms STRINGH and g:Profiler, respectively.

The *Artemia salina* (brine shrimp) lethality test was performed to define the biocompatibility limits of the present extracts. This test is widely used as an alternative toxicological model to predict the toxicity limits in eukaryotic cells [72]. In this regard, shrimp nauplii were exposed to the extracts for 24 h (0.1–20 mg/mL), showing LC_50_ values > 3 mg/mL. Considering these results, a concentration that was at least ten-fold lower was chosen for the pharmacological assays conducted on the human prostate cancer PC3 cell line, which was exposed to the extracts (100–300 µg/mL) to evaluate antiproliferative effects. This is consistent with the study by Stolarczyk and colleagues [42] that showed antiproliferative effects induced by *E. hirsutum* in hormone-dependent prostate cancer cells (LNCaP). Furthermore, in agreement with our recent finding of antiproliferative effect induced by the water extract of *E. angustifolium* (250 µg/mL) on PC3 cells [73], we observed the reduction of PC3 viability (Figure 1) occurring at the highest tested concentration (300 µg/mL) of methanol and methanol/water extracts of roots and aerial parts of *E. hirsutum*. Despite having a lower potency, the antiproliferative effect induced by this extract concentration in PC3 cells was also similar to that induced by daunorubicin 3.12 µg/mL [74]. By contrast, the water (infusion) and ethyl acetate extracts of the analyzed plant materials were completely ineffective in reducing the viability of the prostate cancer cells. The differences between the *E. angustifolium* and *E. hirsutum* extracts in inducing antiproliferative effects on the same tumoral cell line could be related, albeit partially, to the different pattern of secondary metabolites measured via HPLC analysis. If the *E. angustifolium* water extract was particularly rich in catechins, these flavonoids were not detected in the *E. hirsutum* extracts. On the other hand, the root and aerial part extracts were particularly rich in oenothein B, myricetins, oxo-dihydroxy-octadecenoic acid, and trihydroxy-octadecenoic acid. Therefore, these phytochemicals were selected for a bioinformatics analysis with the aim of predicting the protein targets underlying the antiproliferative effects. As shown in Figure 2, oenothein B, myricetins, oxo-dihydroxy-octadecenoic acid, and trihydroxy-octadecenoic acid were able to interact with a wide number of proteins that are also involved in oxidative stress and inflammation, among which are oxidoreductases and secreted proteins. In detail, myricetins were predicted to interact with cycloxygenase-2 (COX-2) and different cytokines, among which tumor necrosis factor α (TNFα) is present. The interaction between TNFα and oxo-dihydroxy-octadecenoic acid was predicted as well. Considering these results, we also evaluated the gene expression of COX-2 and TNFα in PC3 cells after exposure to the methanol and methanol/water extracts of aerial parts and the methanol/water root extract, finding a significant inhibition of the gene expression of both COX-2 and TNFα (Figure 3A,B). These effects were paralleled by the decreased gene expression of interleukin-8 (IL-8) and NFkB (Figure 4A,B) in the same experimental condition, whereas the inhibition of prostaglandin E2 (PGE2) release (Figure 5) by PC3 cells supports the reduction of COX-2 gene expression as one of the anti-inflammatory mechanisms exerted by the present extracts. However, all of the *E. hirsutum* extracts were able to increase the gene expression of IL-6 (Figure 6), and this is apparently in contrast with the aforementioned anti-inflammatory properties. However, Eskandari et al. [75] showed that the inhibition of PC3 cell viability induced by ellagic acid was mirrored by the concomitant increase of IL-6 gene expression and release. This is also consistent with the dual role of IL-6, which can exert both pro-inflammatory and anti-inflammatory effects, depending upon tissue and experimental condition [76]. Finally, considering the strict relationships between cancer and inflammation, especially in prostate cancer [77], we also measured the gene expression of TIMP-1 and VEGFA, which are deeply involved in inflammation and cancer [78]. Specifically, TIMP-1 and VEGFA are reported to influence angiogenesis in opposite ways, with inhibitory and stimulatory effects, respectively [78]. The tested extracts were effective in inhibiting the gene expression of VEGFA and TIMP-1 (Figure 7A,B) as well. Regarding the inhibition of VEGFA gene expression, the present results agree with the literature data, strongly indicating the role of this protein as angiogenetic and tumor prognostic factor [79]. On the other hand, the reduction of TIMP1 gene expression is apparently in contrast with the antiproliferative effect on PC3 cells. TIMP1 is known to inhibit angiogenesis in cancer [78]. However, it is also true that TIMP1 gene expression is induced by pro-inflammatory cytokines [80]. In this context, we cannot exclude that the reduction of TIMP1 gene expression following extract treatment could be related, albeit partially, to the inhibition of the gene expression of the cytokines tested in the present experimental paradigm. Intriguingly, the the bioinformatics platform STRINGH showed functional interactions between all of the assayed proteins (Figure 8A), whereas the KEGG analysis indicated the probable (*p* < 0.05) modulation of different pathways, involved in cancer and inflammation, in the PC3 cells exposed to the extracts (Figure 8B). Overall, the present findings demonstrate the anti-inflammatory and antiproliferative effects of *E. hirsutum* against human prostate cancer cells, which deserve further investigations with independent experimental models, including in vivo studies.

## 4. Conclusions

The chemical profile and biological properties of various extracts of *E. hirsutum* indicated that the plant possessed unique properties. Based on the HPLC-ESI-MS^n^ results, the extracts were rich in phenolic compounds, especially in oenothein B and myricetin derivatives. In particular, the methanol extract of the aerial parts and roots had the greatest antioxidant potentials in the assays that were conducted. However, different results were obtained for each assay with respect to enzyme inhibitory abilities. In the PC3 cancer assay, the methanol and methanol/water extracts of both parts exhibited good reduction on the growth of cancer cells. The pattern of gene expression following PC3 cell exposure to the present extracts also pointed to anti-inflammatory effects that could explain, albeit partially, the antiproliferative effects. In this context, the present findings further strengthen the potential use of plants belonging to the genus *Epilobium* for the management of the burden of inflammation and oxidative stress occurring in lower urinary tract diseases, among which prostatitis is included [73,81]. The results obtained here can have an impact on understanding the rational phytotherapy use of *E. hirsutum*.

## Figures and Tables

**Table 1:** Total phenolic and flavonoid content of *Epilobium hirsutum*.

Parts	Solvents	TPC (mg GAE/g)	TFC (mg RE/g)
Aerial parts	EA	43.52 ± 1.09 ^d^	10.49 ± 0.17 ^d^
	MeOH	254.55 ± 0.72 ^a^	87.66 ± 0.38 ^a^
	Infusion	211.37 ± 1.98 ^b^	27.59 ± 4.17 ^c^
	MeOH/Water	202.97 ± 2.19 ^c^	41.80 ± 0.66 ^b^
Roots	EA	32.52 ± 0.40 ^d^	6.36 ± 0.15 ^a^
	MeOH	109.16 ± 0.05 ^c^	2.54 ± 0.16 ^c^
	Infusion	152.64 ± 1.25 ^b^	3.71 ± 0.94 ^b^
	MeOH/Water	156.27 ± 0.95 ^a^	2.41 ± 0.07 ^c^

Values are reported as the mean ± S.D. of three parallel measurements. TPC: total phenolic content; TFC: total flavonoid content; GAE: gallic acid equivalent; RE: rutin equivalent. Different letters in the same column for each plant part indicate significant differences in the tested extracts (*p* < 0.05).

**Table 2:** Characterization of the compounds found in the analyzed extracts of *Epilobium hirsutum*.

No.	t*_R_* (min)	[M-H]^−^ *m/z*	*m/z* (% Base Peak)	Assigned Identification	Aerial Parts	Roots
1	1.8	377	MS^2^ [377]: 341 (100), 179 (55), 161 (23) MS^3^ [377→341]: 179 (100), 119 (35), 113 (9), 101 (11)	Disaccharide (HCl adduct)	MeOH MEOH:H_2_O	MeOH MEOH:H_2_O EA
2	1.8	195	MS^2^ [195]: 177 (41), 159 (15), 129 (100)	Gluconic acid	Inf	Infusion MEOH:H_2_O
3	2.1	191	MS^2^ [191]: 173 (37), 111 (100)	Isocitric acid	All	--
4	2.3	783 [M-2H]^2−^	MS^2^ [783]: 935 (26), 765 (100), 698 (6), 633 (3) MS^3^ [783→765]: 765 (100), 615 (4), 597 (32), 301 (20), 275 (8)	Oenothein B isomer	MeOH MEOH:H_2_O	MeOH MEOH:H_2_O EA
5	2.6	191	MS^2^ [191]: 173 (28), 111 (100)	Citric acid	All	İnfusion
6	3.1	483	MS^2^ [483]: 331 (100), 193 (14), 169 (58) MS^3^ [483→331]: 193 (54), 169 (100)	Digalloylglucose	Inf MEOH:H_2_O	MeOH İnfusion MEOH:H_2_O
7	3.3	169	MS^2^ [169]: 125 (100)	Gallic acid	All	All
8	3.8	483	MS^2^ [483]: 331 (100), 169 (52) MS^3^ [483→331]: 193 (100), 169 (100)	Digalloylglucose	MeOH Inf MEOH:H_2_O	MeOH İnfusion MEOH:H_2_O
9	5.3	299	MS^2^ [299]: 179 (100), 161 (38), 143 (44), 119 (98), 113 (44), 101 (56)	Unknown	All	--
10	6.0	311	MS^2^ [311]: 179 (54), 149 (100), 135 (6)	Caftaric acid	MeOH Inf MEOH:H_2_O	--
11	7.7	783 [M-2H]^2−^	MS^2^ [783]: 935 (25), 765 (100), 698 (7) MS^3^ [783→765]: 765 (100), 597 (14), 301 (16)	Oenothein B isomer	MeOH Inf MEOH:H_2_O	All
12	8.8	163	MS^2^ [163]: 119 (100)	Coumaric acid	MeOH Inf MEOH:H_2_O	Inf
13	10.3	325	MS^2^ [325]: 193 (100) MS^3^ [325→193]: 178 (69), 149 (100), 134 (57)	Ferulic acid pentoside	MeOH Inf MEOH:H_2_O	MeOH Inf MEOH:H_2_O
14	10.5	431	MS^2^ [431]: 385 (100), 223 (11) MS^3^ [431→385]: 223 (82), 205 (45), 179 (16), 161 (36), 153 (100)	Roseoside (formate adduct)	MeOH Inf MEOH:H_2_O	MeOH EA
15	11.7	785	MS^2^ [785]: 765 (60), 633 (66), 483 (100), 301 (24)	hexahydroxydiphenoyl-digalloyl-glucose	MeOH Inf MEOH:H_2_O	MeOH Inf MEOH:H_2_O
16	13.0	635	MS^2^ [635]: 465 (100) MS^3^ [635→465]: 313 (100), 235 (14), 169 (45)	Trigalloylglucose	MeOH MEOH:H_2_O	MeOH MEOH:H_2_O
17	17.1	479	MS^2^ [479]: 317 (100), 316 (95) MS^3^ [479→317]: 271 (94), 179 (100), 151 (36)	Myricetin-*O*-hexoside	All	All
18	19.7	449	MS^2^ [449]: 317 (43), 316 (100) MS^3^ [449→316]: 271 (100), 179 (39), 151 (32)	Myricetin-*O*-pentoside	All	All
19	20.1	463	MS^2^ [463]: 317 (66), 316 (100) MS^3^ [463→316]: 271 (100), 179 (34), 151 (26)	Myricetin-*O*-deoxyhexoside	All	All
20	20.9	463	MS^2^ [463]: 301 (100) MS^3^ [463→301]: 255 (32), 179 (86), 151 (100)	Quercetin-*O*-hexoside	All	MeOH EA
21	23.5	447	MS^2^ [447]: 285 (100), 255 (27) MS^3^ [447→285]: 257 (8), 255 (100), 227 (10)	Kaempferol.-*O*-hexoside	All	MeOH EA
22	23.5	433	MS^2^ [433]: 301 (100) MS^3^ [433→301]: 271 (45), 179 (100), 151 (75)	Quercetin-*O*-pentoside	All	MeOH EA MEOH:H_2_O
23	24.3	433	MS^2^ [433]: 301 (100) MS^3^ [433→301]: 271 (26), 255 (19), 179 (100), 151 (40)	Quercetin-*O*-pentoside	All	EA
24	24.8	447	MS^2^ [447]: 301 (100) MS^3^ [447→301]: 179 (75), 151 (100)	Quercetin-*O*-deoxyhexoside	All	All
25	26.1	417	MS^2^ [417]: 285 (70), 284 (100) MS^3^ [417→284]: 255 (100)	Kaempferol-*O*-pentoside	All	MeOH EA
26	26.9	317	MS^2^ [317]: 179 (100), 151 (35)	Myricetin	MeOH EA MEOH:H_2_O	--
27	28.9	431	MS^2^ [431]: 285 (100) MS^3^ [431→285]: 255 (100)	Kaempferol-*O*-deoxyhexoside	All	EA
28	29.6	711	MS^2^ [711]: 665 (62), 503 (100) MS^3^ [711→503]: 485 (100), 453 (30), 441 (60), 409 (32)	Saponin	All	All
29	32.3	711	MS^2^ [711]: 665 (75), 503 (100) MS^3^ [711→503]: 485 (100), 441 (15)	Saponin	All	All
30	35.5	301	MS^2^ [301]: 179 (100), 151 (99)	Quercetin	MeOH EA MEOH:H_2_O	--
31	39.0	327	MS^2^ [327]: 291 (56), 229 (100), 211 (87), 171 (96)	Oxo-dihydroxy-octadecenoic acid	All	All
32	40.2	695	MS^2^ [695]: 649 (62), 487 (100)	Unknown	MeOH Inf MEOH:H_2_O	MeOH Inf MEOH:H_2_O
33	40.5	329	MS^2^ [329]: 311 (23), 293 (31), 229 (100), 211 (98), 171 (78)	Trihydroxy-octadecenoic acid	All	All

**Table 3:** Relative peak areas and heat map of extracts of the aerial parts of *Epilobium hirsutum* *.

Peak	Compound	MeOH	Inf	MeOH:H_2_O	EA
1	Disaccharide	1.01	0.00	0.62	0.00
2	Gluconic acid	0.00	0.55	0.00	0.00
3	Isocitric acid	0.21	0.15	0.78	0.38
4	Oenothein B isomer	3.45	0.00	1.07	0.00
5	Citric acid	0.16	0.05	0.37	0.29
6	Digalloylglucose	0.00	0.51	0.08	0.00
7	Gallic acid	0.13	0.20	1.56	1.41
8	Digalloylglucose	0.16	0.75	0.34	0.00
9	Unknown	1.08	1.86	0.90	1.15
10	Caftaric acid	0.23	1.16	0.34	0.00
11	Oenothein B isomer	13.45	37.99	12.68	0.00
12	Coumaric acid	0.61	2.86	1.03	0.00
13	Ferulic acid pentoside	0.79	3.29	0.87	0.00
14	Roseoside	1.57	0.92	1.06	0.00
15	HHDP-digalloyl-glucose	0.73	0.29	0.77	0.00
16	Trigalloylglucose	0.72	0.00	0.60	0.00
17	Myricetin-*O*-hexoside	21.32	14.43	22.88	7.84
18	Myricetin-*O*-pentoside	15.26	8.80	17.09	18.20
19	Myricetin-*O*-deoxyhexoside	8.80	6.73	9.41	18.59
20	Quercetin-*O*-hexoside	2.56	1.73	2.44	2.38
21	Kaempferol.-*O*-hexoside	1.52	1.23	1.62	1.51
22	Quercetin-*O*-pentoside	3.21	1.80	3.53	2.08
23	Quercetin-*O*-pentoside	2.60	1.20	2.12	3.90
24	Quercetin-*O*-deoxyhexoside	5.82	3.61	5.26	16.55
25	Kaempferol-*O*-pentoside	1.46	0.75	1.04	1.27
26	Myricetin	2.12	0.00	1.53	1.10
27	Kaempferol-*O*-deoxyhexoside	0.56	0.28	0.57	1.46
28	Saponin	1.82	1.92	2.09	1.13
29	Saponin	1.21	0.91	1.08	0.91
30	Quercetin	0.68	0.00	0.52	0.74
31	Oxo-dihydroxy-octadecenoic acid	2.59	3.22	2.68	11.80
32	Unknown	2.08	1.01	1.43	0.00
33	Trihydroxy-octadecenoic acid	2.09	1.79	1.66	7.29

* The darker the color indicates the higher the abundance.

**Table 4:** Relative peak areas and heat map of extracts of the roots of *Epilobium hirsutum* *.

Peak	Compound	MeOH	Inf	MeOH:H_2_O	EA
1	Disaccharide	7.38	0.00	1.23	0.24
2	Gluconic acid	0.00	3.08	1.17	0.00
3	Isocitric acid	0.00	0.41	0.00	0.00
4	Oenothein B isomer	4.51	0.00	7.03	1.15
5	Citric acid	0.00	0.00	0.00	0.00
6	Digalloylglucose	0.65	0.89	0.42	0.00
7	Gallic acid	1.05	0.30	3.22	0.38
8	Digalloylglucose	0.69	1.77	1.12	0.00
9	Unknown	0.00	0.00	0.00	0.00
10	Caftaric acid	0.00	0.00	0.00	0.00
11	Oenothein B isomer	43.13	79.93	46.24	2.30
12	Coumaric acid	0.00	0.28	0.00	0.00
13	Ferulic acid pentoside	0.12	1.04	0.46	0.00
14	Roseoside	0.86	0.00	0.00	0.77
15	HHDP-digalloyl-glucose	6.34	0.54	10.71	0.00
16	Trigalloylglucose	3.29	0.00	2.93	0.00
17	Myricetin-*O*-hexoside	2.52	0.45	2.45	2.35
18	Myricetin-*O*-pentoside	2.07	0.20	1.71	2.46
19	Myricetin-*O*-deoxyhexoside	2.26	0.51	2.00	6.39
20	Quercetin-*O*-hexoside	0.23	0.00	0.00	1.38
21	Kaempferol.-*O*-hexoside	0.20	0.00	0.00	0.52
22	Quercetin-*O*-pentoside	0.16	0.00	0.24	0.67
23	Quercetin-*O*-pentoside	0.00	0.00	0.00	0.82
24	Quercetin-*O*-deoxyhexoside	0.56	0.10	0.39	3.58
25	Kaempferol-*O*-pentoside	0.13	0.00	0.00	0.44
26	Myricetin	0.00	0.00	0.00	0.00
27	Kaempferol-*O*-deoxyhexoside	0.00	0.00	0.00	0.66
28	Saponin	3.52	1.45	3.26	6.14
29	Saponin	1.85	0.72	1.61	3.76
30	Quercetin	0.00	0.00	0.00	0.00
31	Oxo-dihydroxy-octadecenoic acid	7.12	4.50	5.18	27.11
32	Unknown	4.47	0.55	3.53	9.27
33	Trihydroxy-octadecenoic acid	6.89	3.28	5.11	29.62

* The darker the color indicates the higher the abundance.

**Table 5:** Antioxidant properties of *Epilobium hirsutum* extracts.

Parts	Solvents	DPPH (mg TE/g)	ABTS (mg TE/g)	CUPRAC (mg TE/g)	FRAP (mg TE/g)	PBD (mmol TE/g)	MCA (mg EDTAE/g)
Aerial parts	EA	74.54 ± 1.02 ^d^	118.95 ± 0.88 ^d^	83.28 ± 2.58 ^d^	37.43 ± 1.53 ^d^	3.11 ± 0.23	43.25 ± 0.97 ^c^
	MeOH	798.89 ± 0.71 ^a^	1382.08 ± 0.81 ^a^	1642.42 ± 5.42 ^b^	677.83 ± 6.13 ^b^	6.80 ± 0.32	63.31 ± 0.79 ^a^
	Infusion	751.13 ± 2.73 ^c^	1254.58 ± 5.10 ^b^	1819.57 ± 65.86 ^a^	756.63 ± 8.28 ^a^	4.72 ± 0.15	58.19 ± 0.24 ^b^
	MeOH/Water	778.54 ± 2.74 ^b^	1078.21 ± 9.41 ^c^	1270.20 ± 16.43 ^c^	573.81 ± 20.77 ^c^	4.25 ± 0.01	37.69 ± 3.15 ^d^
Roots	EA	59.40 ± 0.92 ^d^	114.75 ± 0.18 ^c^	105.14 ± 7.37 ^d^	46.03 ± 1.79 ^d^	2.08 ± 0.32	25.89 ± 4.62 ^c^
	MeOH	316.60 ± 0.40 ^b^	551.11 ± 0.57 ^b^	635.13 ± 11.15 ^c^	218.59 ± 9.91 ^c^	3.81 ± 0.19	39.40 ± 1.40 ^a^
	Infusion	312.62 ± 0.37 ^c^	551.40 ± 0.42 ^b^	1065.96 ± 19.02 ^a^	424.87 ± 3.33 ^a^	3.93 ± 0.01	45.22 ± 1.17 ^a^
	MeOH/Water	317.72 ± 0.11 ^a^	552.33 ± 0.29 ^a^	845.00 ± 29.67 ^b^	376.40 ± 8.80 ^b^	3.76 ± 0.05	44.01 ± 1.95 ^ab^

Values are reported as the mean ± S.D. of three parallel measurements. TE: Trolox equivalent; EDTAE: EDTA equivalent. Different letters in the same column for each plant part indicate significant differences in the tested extracts (*p* < 0.05).

**Table 6:** Enzyme inhibitory properties of *Epilobium hirsutum* extracts.

Parts	Solvents	AChE (mg GALAE/g)	BChE (mg GALAE/g)	Tyrosinase (mg KAE/g)	α-Amylase (mmol ACAE/g)	α-Glucosidase (mmol ACAE/g)
Aerial parts	EA	2.69 ± 0.25 ^b^	4.72 ± 0.35 ^a^	90.24 ± 3.48 ^b^	0.95 ± 0.09 ^a^	1.57 ± 0.04 ^b^
	MeOH	4.48 ± 0.35 ^a^	2.34 ± 0.46 ^b^	106.68 ± 2.02 ^a^	1.02 ± 0.08 ^a^	1.62 ± 0.03 ^a^
	Infusion	na	1.68 ± 0.10 ^c^	49.80 ± 1.54 ^c^	0.17 ± 0.01 ^c^	na
	MeOH/Water	2.76 ± 0.11 ^c^	1.11 ± 0.18 ^d^	103.84 ± 1.27 ^a^	0.73 ± 0.13 ^b^	na
Roots	EA	4.35 ± 0.36 ^a^	5.18 ± 0.44 ^a^	79.30 ± 1.18 ^b^	0.62 ± 0.12 ^b^	1.66 ± 0.01 ^c^
	MeOH	3.00 ± 0.57 ^b^	4.94 ± 0.69 ^ab^	98.31 ± 3.23 ^a^	0.83 ± 0.06 ^a^	1.86 ± 0.01 ^b^
	Infusion	1.01 ± 0.06 ^c^	4.14 ± 0.59 ^b^	34.47 ± 1.09 ^c^	0.14 ± 0.01 ^c^	1.96 ± 0.01 ^a^
	MeOH/Water	2.81 ± 0.15 ^b^	4.23 ± 0.13 ^ab^	96.84 ± 0.48 ^a^	0.61 ± 0.06 ^b^	na

Values are reported as the mean ± S.D. of three parallel measurements. GALAE: galatamine equivalent; KAE: kojic acid equivalent; ACAE: acarbose equivalent; na: not active. Different letters in the same column for each plant part indicate significant differences in the tested extracts (*p* < 0.05).

## Data Availability

All data is contained within the article and Supplementary Materials.

## Supplementary Materials

The following are available online: https://www.mdpi.com/article/10.3390/antiox10091389/s1. Figure S1: Base peak chromatogram of the methanol extract of the aerial parts of *Epilobium hirsutum.*
