# Supplementary material for: Shedding Light into the Connection between Chemical Components and Biological Effects of Extracts from Epilobium hirsutum: Is It a Potent Source of Bioactive Agents from Natural Treasure?
Source: Antioxidants (Basel). 2021 Aug 30;10(9):1389. doi: 10.3390/antiox10091389 (PMC8468522; doi:10.3390/antiox10091389)
Supplement: Supplementary file 1 [file antioxidants-10-01389-s001.zip › antioxidants-1333598-supplementary.pdf]

Article

Shedding Light into the Connection between Chemical Components and Biological Effects of Extracts from *Epilobium hirsutum*: Is It a Potent Source of Bioactive Agents from Natural Treasure?

Gunes Ak[1], Gokhan Zengin[1*], Mohamad Fawzi Mahomoodally[2], Eulogio J. Llorent-Martínez[3] Giustino Orlando[4], Annalisa Chiavaroli[4], Luigi Brunetti[4], Lucia Recinella[4], Sheila Leone[4], Simonetta Cristina Di Simone[4], Luigi Menghini[4*], Claudio Ferrante[4]

[1]Physiology and Biochemistry Research Laboratory, Department of Biology, Science Faculty, Selcuk University, Konya, Turkey; gokhanzengin@selcuk.edu.tr (G.Z.); akguneselcuk@gmail.com (G.A.)

[2]Department of Health Sciences, Faculty of Medicine and Health Sciences, University of Mauritius, Réduit, Mauritius; f.mahomoodally@uom.ac.mu (M.F.M.)

[3]Department of Physical and Analytical Chemistry, University of Jaén, Campus Las Lagunillas S/N, E-23071 Jaén, Spain; ellorent@ujaen.es (E.J.L.M.)

[4]Department of Pharmacy, Botanic Garden "Giardino dei Semplici", Università degli Studi "Gabriele d'Annunzio", via dei Vestini 31, 66100 Chieti, Italy; luigi.menghini@unich.it (L.M.); claudio.ferrante@unich.it (C.F.); annalisa.chiavaroli@unich.it (A.Ch.); disimonesimonetta@gmail.com (S.C.D.S); giustino.orlando@unich.it (G.O.); luigi.brunetti@unich.it (L.B.); sheila.leone@unich.it (S.L.); lucia.recinella@unich.it (L.R.); luigi.menghini@unich.it (L.M.); claudio.ferrante@unich.it (C.F.)

*Correspondence: : gokhanzengin@selcuk.edu.tr (Prof. Gokhan Zengin) and luigi.menghini@unich.it (Prof. Luigi Menghini)

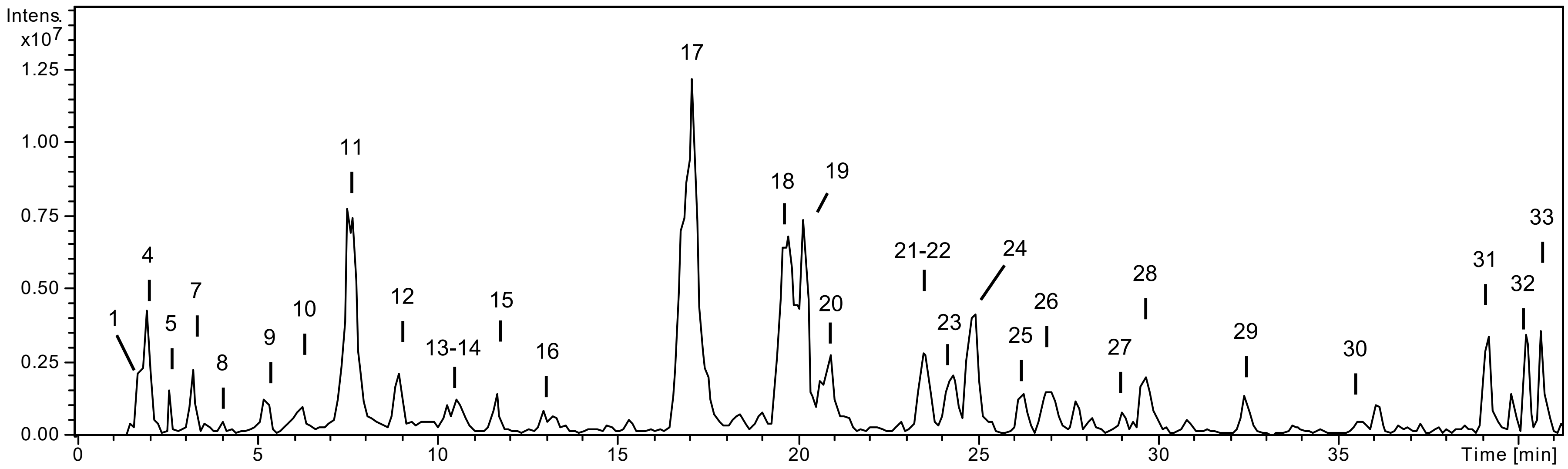

Figure S1. Base peak chromatogram of the methanol extract of aerial parts of *Epilobium hirsutum*.
